# A Green Extraction Process for Polyphenols from Elderberry (*Sambucus nigra*) Flowers Using Deep Eutectic Solvent and Ultrasound-Assisted Pretreatment

**DOI:** 10.3390/molecules25040921

**Published:** 2020-02-19

**Authors:** Olga Kaltsa, Achillia Lakka, Spyros Grigorakis, Ioanna Karageorgou, Georgia Batra, Eleni Bozinou, Stavros Lalas, Dimitris P. Makris

**Affiliations:** 1 Department of Food Science & Nutrition, School of Agricultural Sciences, University of Thessaly, N. Temponera Street, 43100 Karditsa, Greece; okaltsa@uth.gr (O.K.); ACHLAKKA@uth.gr (A.L.); ikarageorgou@uth.gr (I.K.); gbatra@uth.gr (G.B.); empozinou@uth.gr (E.B.); slalas@uth.gr (S.L.); 2 Food Quality & Chemistry of Natural Products, Mediterranean Agronomic Institute of Chania (M.A.I.Ch.), International Centre for Advanced Mediterranean Agronomic Studies (CIHEAM), P.O. Box 85, 73100 Chania, Greece; grigorakis@maich.gr

**Keywords:** deep eutectic solvents, elderberry flowers, extraction, polyphenols, *Sambucus nigra*, ultrasonication

## Abstract

*Sambucus nigra* flowers, known as elderberry flowers (EBF), are a plant tissue rich in polyphenolic phytochemicals with important bioactivities. However, there are few studies dealing with the production of polyphenol-containing EBF extracts. The objective of the investigation presented herein was the development of a high-performance green extraction methodology, to generate EBF extracts enriched in polyphenolic substances, using an efficient deep eutectic solvent, combined with ultrasonication pretreatment. The DES was composed of L-lactic acid (hydrogen bond donor—HBD) and glycine (hydrogen bond acceptor—HBA) and, after an initial screening to properly regulate HBD/HBA ratio, the extraction was optimized by deploying response surface methodology. Under the optimized conditions, which were DES/water (85% *w*/*v*), liquid-to-solid ratio 60 mL g^−1^, and stirring speed 200 rounds per minute, the extraction yield in total polyphenols amounted to 121.24 ± 8.77 mg gallic acid equivalents per g dry matter. The integration of ultrasonication prior to the batch stirred-tank extraction boosted polyphenol recovery of up to 174.73 ± 2.62 mg gallic acid equivalents per g dry matter. Liquid chromatography–mass spectrometry analysis showed that the richest EBF extract obtained was dominated by rutin, a di-p-coumaroylquic acid and chlorogenic acid.

## 1. Introduction

Edible flowers have been used in culinary practice since antiquity, serving not only as food ingredients but also as agents of herbal folk medicine. At present, edible flowers are becoming increasingly popular and, despite being considered a niche market, there has been significant recent attention to edible flower products, raised by evidence concerning their potential as a source of bioactive compounds [1]. In fact, edible flowers may contain a wide variety of phytochemicals, mostly phenolic acids and flavonoids, and exhibit a multitude of biological effects, including antioxidant anti-inflammatory activity, as well as chemopreventive and neuroprotective properties [2]. Several studies have affirmed that flower extracts from a broad spectrum of botanical species may bear a high load of total polyphenols, accompanied by proportional antioxidant activity [3].

Elderberry plant (*Sambucus nigra*) has been used in the treatment of several ailments, and its medicinal properties have been associated with the presence of polyphenols. Elderberry flowers, in particular, may contain significant amounts of flavonols and phenolic acids, such as derivatives of caffeic and p-coumaric acid, chlorogenic and neochlorogenic acids, etc. [4]. By virtue of their polyphenolic richness, elderberry flowers are considered an important polyphenol source with powerful antioxidant activity [5].

Currently, the majority of the extraction processes implemented on industrial scale for the production of cosmetic, pharmaceutical, food ingredients, and fine chemicals is based on solvents of petroleum origin. However, the shift to eco-friendly, bio-based prospects dictates the use of alternative solvents with a green profile, which can be obtained from renewable resources at low cost. The ideal candidates should possess high-dissolving capacity for specific target-molecules and low toxicity; they should be easily biodegradable and recycled without any deleterious environmental impact. The search for liquids that could meet such requirements is an intriguing concept, yet the decision for the use of the most suitable solvent would be a compromise depending on several factors, such as the solute-to-be-recovered, overall process, cost, availability, etc. [6].

Deep eutectic solvents (DES) are tailor-made liquids, which may be easily synthesized using food-grade, inexpensive components, such as polyols, sugars, organic acids, organic acid salts, amino acids, etc. [7]. Over the past few years, DES have gained significant attention because of their high prospect as green solvents, since they are characterized by almost complete absence of toxicity, recyclability, and biodegradability [8]. On the other hand, the flexibility in their synthesis makes possible to tune their composition and therefore their physicochemical properties as desired, lending them a high degree of applicability in processes such as natural product extraction [9].

Ultrasound-assisted extraction (UAE) is highly regarded as a sustainable means of recovering polyphenolic substances from plant material. It requires a rather moderate investment of solvent and energy, and it is also easy to handle, safe, cost-effective, and reproducible. Further important advantages of UAE are the operation under conditions of atmospheric pressure and temperature [10]. UAE involves acoustic cavitation, which may bring about cell walls disruption, thereby favoring the release of bioactive compounds, and can be very effectively applied to obtain polyphenolic phytochemicals [11]. Recent evidence pointed out that UAE may not be very effective as a standalone method, but high extraction yields could be achieved by a combination of UAE and stirred-tank extraction [12]. The following studies concurred with these findings, yet the effect of ultrasonication as a pretreatment step was somewhat unclear, exhibiting dependence on temperature [13,14]. Thus, additional information is necessary to further clarify this issue and bring out the usefulness of ultrasonication in sample pretreatment.

On this basis, EBF were chosen as a plant material with peculiar polyphenolic composition, and samples were ultrasonication-pretreated before subjected to stirred-tank extraction with a highly efficient DES, composed of L-lactic acid and glycine. The extracts obtained were examined regarding their polyphenolic load but also antioxidant properties, and further extract evaluation was carried out by analyzing the polyphenolic profile with liquid chromatography–mass spectrometry.

## 2. Materials and Methods

### 2.1. Chemicals

Gallic acid hydrate was from Panreac (Barcelona, Spain). Glycine (99.5%) was from Applichem (Darmstadt, Germany). Iron chloride hexahydrate was from Merck (Darmstadt, Germany). L-Lactic acid, ascorbic acid (99.5%), sodium acetate trihydrate, sodium carbonate anhydrous (99%) and aluminum chloride anhydrous (98%) were from Penta (Praha, Czechia). Chlorogenic acid (95%) was from Fluorochem (Hadfield, UK). Neochlorogenic acid (≥98%) was from Merck (Darmstadt, Germany). Folin-Ciocalteu reagent, rutin (quercetin 3-*O*-rutinoside) hydrate (>94%), isorhamnetin 3-*O*-glucoside and 2,4,6-tris(2-pyridyl)-*s*-triazine (TPTZ) were from Sigma-Aldrich (St. Louis, MO, USA).

### 2.2. Plant Material and Handling

Elderberry (*Sambucus nigra*) flowers (EBF) were collected during summer 2019 from the area of Neohori (Domokos, Central Greece, altitude 760 m, latitude 39.03682o, longitude 22.51955o), from a producer that utilizes certified botanical material. Further identification of the specimen was obtained from the Mediterranean Plant Conservation Center, (Chania, Greece). The plant tissue was freeze dried using a Telstar Cryodos 80 freeze dryer (Telstar Industrial, S.A., Terrassa, Spain) for 12 h, and then ground in a ball-mill to yield a pulverized material with 0.284 mm approximate average particle diameter. The material was stored in plastic containers, at 4 °C, until used.

### 2.3. Synthesis of the Deep Eutectic Solvent (DES)

A series of DES composed of L-lactic acid (hydrogen bond donor—HBD) and glycine (hydrogen bond acceptor—HBA) was synthesized, based on a previous method [15]. Accurately weighted amounts of both HBD and HBA were placed into a round-bottom glass flask and heated moderately (80 °C) in oil bath for approximately 120 min, until the formation of a perfectly transparent liquid. Heating was provided by a temperature-cotrolled hotplate (Witeg, Wertheim, Germany). The DES was allowed to cool down to ambient temperature and stored in a sealed vial, in the dark. The appearance of crystals that would indicate DES instability was visually inspected at regular intervals over 6 weeks.

### 2.4. Batch Extraction Process

Exact mass of 0.570 g of dried EBF was introduced into a 50 mL round-bottom flask and mixed with 20 mL of solvent to yield a liquid-to-solid ratio (R_L/S_) of 35 mL g^−1^. Extraction was performed for 150 min in oil bath, under constant heating (50 °C) and stirring (500 rpm), provided by a temperature-cotrolled hotplate (Witeg, Wertheim, Germany). All DES were tested as 70% (*w*/*v*) aqueous mixtures. Control extractions with deionized water, 60% (*v*/*v*) aqueous ethanol, and 60% (*v*/*v*) aqueous methanol were also performed. Extracts were centrifuged at 10,000× *g* for 10 min before further analyses.

### 2.5. Ultrasound-Assisted Pretreatment

The pretreatment was delivered as described elsewhere [13], with some minor modifications. An Elma D-78224 Singen HTW heated ultrasonic bath (Elma Schmidbauer GmbH, Singen, Germany), operated at a frequency of 50 Hz and a power of 550 W, was fed with 7.3 L deionized water to provide an acoustic energy density of 75.3 W L^−1^. Sample volume of 20 mL was placed in a 25 mL Duran™ glass bottle, immersed into the ultrasonic bath and irradiated for varying resident time, at ambient temperature (22 ± 1 °C).

### 2.6. Experimental Design and Deployment of Response Surface Methodology

Details of the experimental design employed have been described elsewhere [13]. Briefly, the experimental set-up was based on a Box-Behnken design with three central points. The three independent variables chosen were the concentration of DES in aqueous mixtures (*C*_DES_, %*w*/*v*), the liquid-to-solid ratio (R_L/S_, mL g^−1^) and the stirring speed (S_S_, rpm). Codified and actual levels of the variables are analytically given in Table 1. Appraisal of model fitting was based on ANOVA and lack-of-fit test.

### 2.7. Total Polyphenol Determination

A Folin–Ciocalteu protocol developed previously was used [16]. A 1:50 dilution of samples with 0.5% aqueous formic acid was performed prior to determinations, and then, 0.1 mL of diluted sample and 0.1 mL Folin–Ciocalteu reagent were pipetted into a 1.5 mL Eppendorf tube. Following a 2 min reaction, 0.8 mL of sodium carbonate (5% *w*/*v*) was added, and the mixture was incubated for 20 min in a water bath, at 40 °C. Total polyphenol concentration (*C*_TP_) was determined by the absorbance at 740 nm, using a gallic acid calibration (10–80 mg L^−1^). Yield in total polyphenols was calculated as mg gallic acid equivalents (GAE) per g dry mass (dm) [17].

### 2.8. Total Flavonoid Determination

A methodology previously reported was used [18]. Samples were suitably diluted with deionized water, and 0.1 mL of each sample was mixed with 0.86 mL 35% (*v*/*v*) aqueous ethanol and 0.04 mL of reagent containing of 5% (*w*/*v*) aluminum chloride and 0.5 M sodium acetate. Samples were left to react for 30 min at ambient temperature before reading the absorbance at 415 nm. Rutin was used as the calibration standard and 15–300 mg L^−1^ and yield in total flavonoids (Y_TFn_) was estimated as mg rutin equivalents (RtE) per g dm.

### 2.9. Determination of the Antiradical Activity (A_AR_)

The radical-scavenging activity was estimated with a DPPH assay [19]. Volume of 0.025 mL of sample, previously diluted 1:50 with methanol, was combined with 0.975 mL DPPH (100 μM in methanol) at room temperature. Absorbance was obtained at 515 nm, at *t* = 0 min (immediately after mixing) and at *t* = 30 min. The A_AR_ of the extract was then determined using the following equation:

(1) $$A_{\mathrm{AR}}\;=\;\frac{C_{\mathrm{DPPH}}}{C_{\mathrm{TP}}}\times \left(1-\frac{A_{515\left(f\right)}}{A_{515\left(i\right)}}\right)\times Y_{\mathrm{TP}}$$

where *C*_DPPH_ represents the DPPH concentration (μM) and *C*_TP_ the total polyphenol concentration (mg L^-1^) in the reaction mixture; A_515(f)_ is the A_515_ at *t* = 30 min and A_515(i)_ the A_515_ at *t* = 0; and Y_TP_ is the extraction yield (mg g^-1^) in total polyphenols. A_AR_ was given as μmol DPPH g^−1^ dm.

### 2.10. Determination of the Reducing Power (P_R_)

The ferric-reducing power was assayed as previously described [20]. All samples were diluted 1:50 and 0.05 mL of each sample was incubated with 0.05 mL FeCl_3_ (4 mM in 0.05 M HCl), in a water bath, for 30 min, at 37 °C. Then 0.9 mL of TPTZ solution (1 mM in 0.05 M HCl) was added and samples were allowed to stand for 10 min, at room temperature. Absorbance readings were accomplished at 620 nm and P_R_ was computed as μmol ascorbic acid equivalents (AAE) g^−1^ dm, using a calibration curve constructed with freshly prepared ascorbic acid (50–300 μM). Results were given as µmol ascorbic acid equivalents (AAE) per g dry mass.

### 2.11. Liquid Chromatography–Diode Array–Mass Spectrometry (LC-DAD-MS)

A modification method previously described was employed [20]. The equipment used was a Finnigan (San Jose, CA, USA) MAT Spectra System P4000 pump, a UV6000LP diode array detector and a Finnigan AQA mass spectrometer. A Fortis RP-18 column, 150 mm × 2.1 mm, 3 μm, at 40 °C, with a 10 μL injection loop was used for all analyses. Electrospray ionization (ESI) in positive ion mode was implemented for mass spectra acquisition, with probe temperature set 250 °C, the source voltage at 25 V, capillary voltage was 4 kV, the acquisition set at 20 and 70 eV, and detector voltage 450 V. The eluents were (A) 2% acetic acid and (B) methanol. The flow rate was 0.3 mL min^−1^, and the elution program used was 0–30 min, 0% to 100% methanol, 30–40 min, 100% methanol.

### 2.12. High-Performance Liquid Chromatography–Diode Array (HPLC-DAD)

Chromatography was performed based on a recently reported methodology [15]. A Shimadzu CBM-20A liquid chromatograph (Shimadzu Europa GmbH, Germany), equipped with a SIL-20AC auto sampler and a CTO-20AC column oven, was used. The detector was a Shimadzu SPD-M20A and system interface was by Shimadzu LC solution software. A Phenomenex Luna C18(2) column (100 Å, 5 μm, 4.6 × 250 mm) (Phenomenex, Inc., USA), maintained at a temperature of 40 °C was used for all analyses. Eluents were (A) 0.5% aqueous formic acid and (B) 0.5% formic acid in MeCN/water (6:4), and the flow rate was 1 mL min^−1^. A 20 μL loop was employed for injecting samples into the HPLC, which were then analyzed by the following elution program: 100% A to 60% A in 40 min, 60% A to 50% A in 10 min, 50% A to 30% A in 10 min, and then isocratic elution for another 10 min. The column was washed with 100% MeCN and re-equilibrated with 100% eluent A before the next injection. Quantification was performed at 320 and 360 nm, for hydroxycinnamates and flavonols, respectively, based on calibration curves (1–50 µg mL^−1^), constructed with neochlorogenic acid (R^2^ = 0.9997), chlorogenic acid (R^2^ = 0.9999), *p*-coumaric acid (R^2^ = 0.9999), rutin (R^2^ = 0.9990) and isorhamnetin 3-*O*-glucoside (R^2^ = 0.9999).

### 2.13. Statistical Analysis

Extractions were carried out at least twice, and determinations at least in triplicate. Values given are means ± standard deviation (SD). Correlations were established with regression analysis, at least at a 95% significance level (*p* < 0.05), using SigmaPlot™ 12.5. The design of experiment and response surface methodology, as well as all associated statistics were done with JMP™ Pro 13.

## 3. Results and Discussion

### 3.1. DES Synthesis and the Effect of HBD:HBA Molar Ratio ($R_{mol}^{D/A}$)

**Figure 1:**
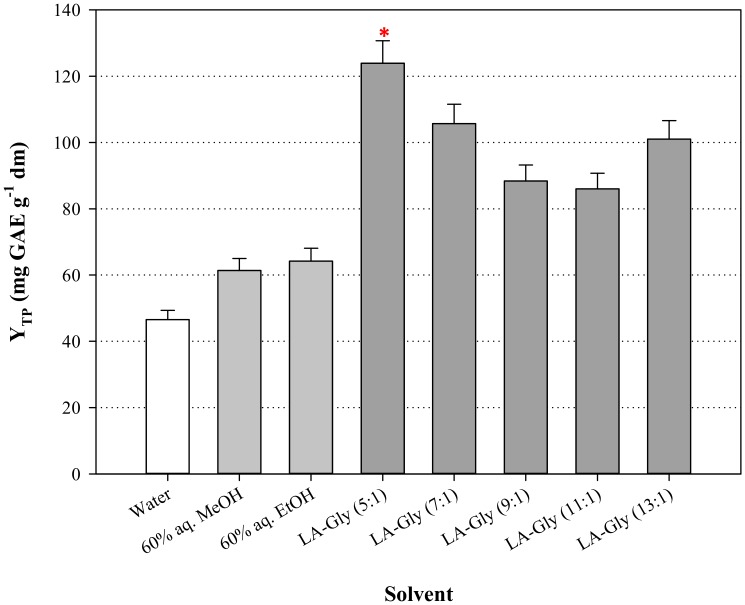
Bar diagram illustrating the effect of the extraction efficiency of the DES tested. Extractions were performed with *C*_DES_ 70% (*w*/*v*), R_L/S_ 35 mL g^−1^, at 50 °C and 500 rpm, for 150 min. Asterisk (*) indicates statistically different value (*p* < 0.05).

The selection of an appropriate $R_{\mathrm{mol}}^{D/A}$ is important in the synthesis of DES because the molar proportion between HBD and HBA may critically affect DES extraction performance [13,14]. Earlier investigations outlined that DES composed of L-lactic acid (LA) and glycine (Gly) were not stable at $R_{\mathrm{mol}}^{D/A}$ ≤ 3 and tended to form plastic solid at room temperature [21]. Following examinations pointed out that stability (no crystallization) of DES composed of LA and Gly could be assured at $R_{\mathrm{mol}}^{D/A}$ ≥ 5 [22]. In a recent study, it was clearly showed that switching $R_{\mathrm{mol}}^{D/A}$ from 5 up to 13, extraction efficiency may be significantly impacted [13]. Thus, in this study, screening of DES with $R_{\mathrm{mol}}^{D/A}$ ranging from 5 to 13, was the first step towards the development of an effective solvent. All DES were tested as 70% (*w*/*v*) aqueous mixtures and the results obtained are presented in Figure 1. The DES with $R_{\mathrm{mol}}^{D/A}$ = 5 was proven to be the highest-performing system, giving significantly increased Y_TP_ (*p* < 0.05).

**Figure 2:**
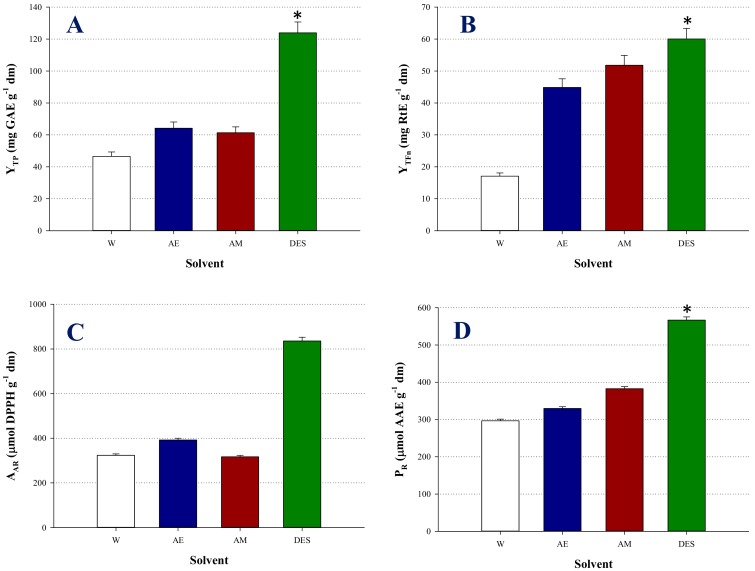
Bar diagrams displaying characteristics of the extracts produced with DES, as compared with control solvents. (**A**), yield in total polyphenols (Y_TP_); (**B**), yield in total flavonoids (Y_TFn_); (**C**), antiradical activity (A_AR_); (**D**), reducing power (P_R_). Extractions were performed with *C*_DES_ 70% (*w*/*v*), R_L/S_ 35 mL g^−1^, at 50 °C and 500 rpm, for 150 min. Asterisk (*) indicates statistically different value (*p* < 0.05).

To obtain a more integrated picture, the efficiency of LA-Gly (5:1) was further appraised by comparing its performance with that of two other green solvents, namely 60% (*v*/*v*) aqueous ethanol and water, but also with a commonly used solvent, 60% (*v*/*v*) aqueous methanol. Apart from Y_TP_, the Y_TFn_, A_AR_, and P_R_ were also considered, and the outcome is depicted in Figure 2. LA-Gly (5:1) gave higher Y_TP_ and Y_TFn_, which were statistically significant (*p* < 0.05) (Figure 2A,B). Furthermore, the EBF extracts obtained with LA-Gly (5:1) had higher, but statistically non-significant (*p* > 0.05) A_AR_ and P_R_, (Figure 2C,D). Considering all these results together, it was concluded that LA-Gly (5:1) was the highest-performic system.

### 3.2. Optimization of Extraction Performance

The experimental design was set up to evaluate the influence of three key extraction variables (*C*_DES_, R_L/S_, S_S_) on the DES performance for polyphenol recovery. The scope was the generation of a polynomial equation (model) based on the experimental data, to deliver a concrete statistical prevision. Validity of the fitted model was assessed by both ANOVA and lack-of-fit tests (Table 2). All non-significant terms were omitted from the equation derived, and thus its final form was the following:

(2) $$Y_{\mathrm{TP}}\;=\;96.38\;+\;3.95X_{2}\;-\;4.82X_{3}\;3.99\;X_{2}X_{3}\;+\;4.48X_{1}^{2}\;+\;6.50X_{2}^{2}\;+\;6.33X_{3}^{2}\;(R^{2}\;=\;0.94,\;p\;=\;0.012)$$

The square correlations coefficient (R^2^) and the *p*-value provide an indication of the total variability around the mean calculated by the model. Since R^2^ was 0.94 and the *p* value (considering a confidence interval of 95%) was highly significant, it could be argued that the model displayed a sound fitting to the experimental data. Measured and predicted Y_TP_ values for each design point are analytically given in Table 3.

**Figure 3:**
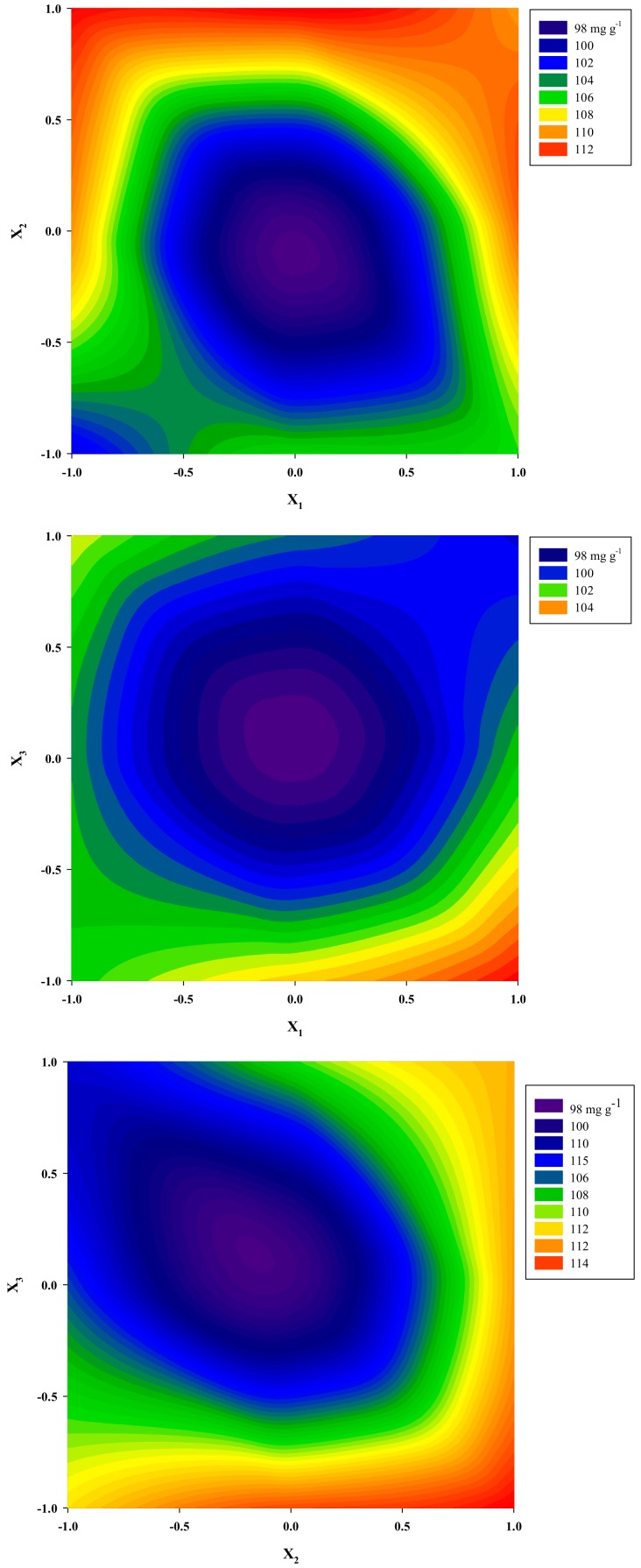
Contour plots showing the effect of independent (process) variables on the response (Y_TP_). Upper, middle, and lower plots correspond to covariation of X_1_ and X_2_, X_1_ and X_3_, and X_2_ and X_3_.

The three-dimensional plots crafted using the model, show at-a-glance variations of the response (Y_TP_) as a function of changes in the three model variables (Figure 3). The use of the desirability function permitted the optimization of the levels of all three variables simultaneously, to achieve maximum system performance and enabled the calculation of the set of conditions that would allow for attaining the highest theoretical yield (121.24 ± 8.77 mg GAE g^−1^ dm). These conditions were *C*_DES_ = 85% (*w*/*v*), R_L/S_ = 60 mL g^−1^ and S_S_ = 200 rpm. Confirmation of the validity of the model was done by carrying out three extractions under the optimal conditions, which gave Y_TP_ of 114.96 ± 5.02.

ANOVA revealed that for *C*_DES_ (X_1_), only the quadratic effect was significant; increasing R_L/S_ (X_2_) had a positive effect on Y_TP_, whereas the effect of S_S_ (X_3_) was negative. No cross effects between process variables were found to be significant, evidence that every variable exerted distinguishable influence on the extraction yield. The optimized predicted *C*_DES_ levels were in line with previous results on polyphenol extraction with DES, suggesting 80% (*w*/*v*) to be the most suitable *C*_DES_ for effective polyphenol recovery [23,24].

Appropriate mixing of DES with water is a key step in regulating critical DES properties, such as viscosity and polarity [25]. Yet, water cannot exceed a certain level because this would provoke DES disintegration and abolishment of its intrinsic characteristics [26].

R_L/S_ is also a parameter that could profoundly affect solid–liquid extraction, since R_L/S_ defines the concentration gradient of the solute (polyphenols) between the solid particles and the liquid phase. This gradient is considered to be the driving force for diffusion, which governs polyphenol entrainment from the inner of the solid to the liquid. Diffusivity may be increased by raising R_L/S_ [27]; however, the optimum R_L/S_ found for polyphenol extractions with DES may vary from 29.5 [28] to as high as 100 mL g^−1^ dm [29,30]. The optimal R_L/S_ determined for EBF (60 mL g^−1^) is in accordance with recent studies on polyphenol extraction with DES from saffron processing wastes (60 mL g^−1^) [15] and hop (59 mL g^−1^) [13].

S_S_ is a variable with crucial role in solid–liquid extraction, and it has been proven that careful S_S_ setting could provide higher extraction yields [27,31]. In a recent study where S_S_ was considered as one of the variables for constructing experimental design, it was found to exert a statistically significant effect on the polyphenol extraction yield [15]. It has been proposed that appropriately set S_S_ may create sufficient turbulence in the extraction tank to increase mass transfer rate. Such an effect has been demonstrated to increase polyphenol diffusivity [27]. On the other hand, optimization of polyphenol extraction has shown that, in some cases, low S_S_ (300 rpm) may favor increased extraction yield, as opposed to higher S_S_ (900 rpm), which apparently was hindering in this regard [30]. In other recent examinations, the findings indicated quite the opposite [15,29]. Since the phenomena associated with the effect of S_S_ may be related with factors such as the nature of the solid material, the solid particle diameter, the solute (polyphenols species), the viscosity of the liquid phase (solvent), etc., the actual effect of S_S_ on extraction yield would be a subject of case experimentation.

### 3.3. Temperature Effects

Extraction temperature is a variable that should be carefully used, because polyphenols are generally considered to be thermosensitive substances. Although, in general, increased temperature may contribute in achieving higher extraction yields, it is not a universal rule that temperature rising generates proportional effect on the extraction yield and antioxidant activity. This argument may be exemplified by results drawn from the extraction of various plant materials, including *Moringa oleifera* leaves [23], onion solid wastes [32,33], chickpea sprouts [34] and red grape pomace [35]. This being the case, the investigation of the effect of temperature on the extraction yield and the antioxidant activity of the extracts merits particular attention.

**Figure 4:**
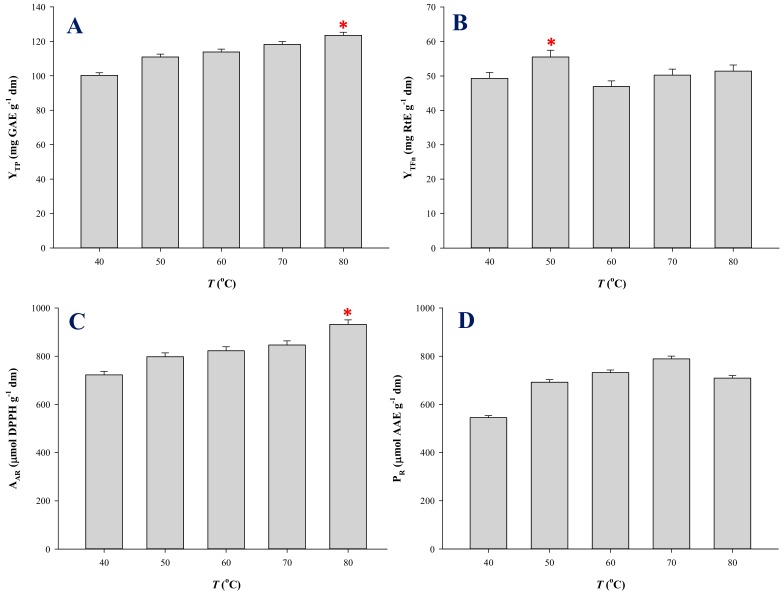
Bar diagram displaying the effect of extraction temperature on the characteristics of the extracts obtained with LA-Gly (5:1), under optimal conditions. (**A**), yield in total polyphenols (Y_TP_); (**B**), yield in total flavonoids (Y_TFn_); (**C**), antiradical activity (A_AR_); (**D**), reducing power (P_R_). Asterisk (*) indicates statistically different value (*p* < 0.05).

Thus, EBF was extracted under optimal conditions, at temperatures ranging from 40 to 80 °C, and the extracts produced were examined by determining Y_TP_, Y_TFn_, A_AR_, and P_R_. Switching temperature from 40 to 80 °C did afford higher Y_TP_, and the value obtained at 80 °C was statistically different (Figure 4A), which pointed emphatically to a strong temperature effect. Likewise, the extracts produced at 80 °C displayed significantly higher A_AR_ (Figure 4C), but for the P_R_, no statistical difference was seen between the levels acquired at 70 and 80 °C (Figure 4D). Contrary to those findings, significantly higher Y_TFn_ was recorded at 50 °C (Figure 4B). The overall picture dictated that extraction temperature up to 80 °C could be used to enrich EBF extracts in polyphenols and enhance their antioxidant activity.

### 3.4. Effect of Ultrasound-Assisted Pretreatment

**Figure 5:**
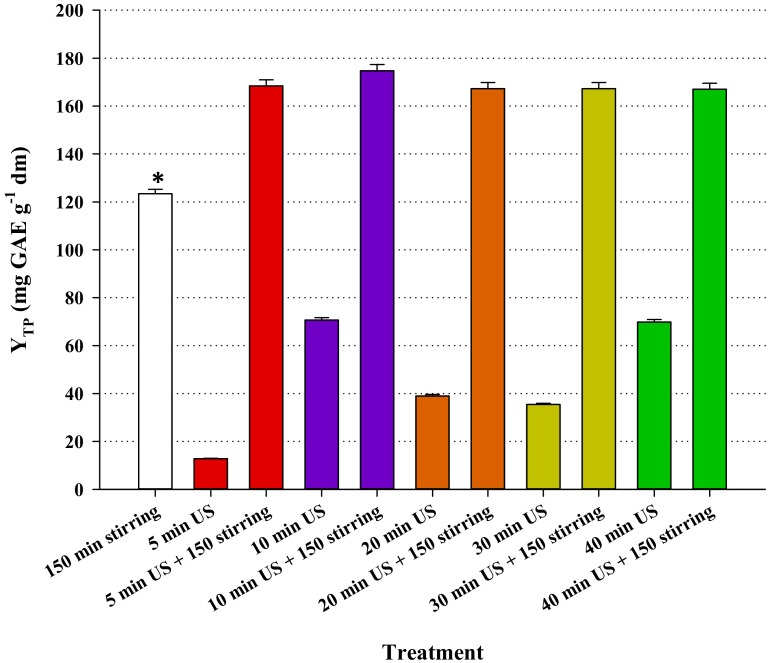
Bar diagram presenting the effect of ultrasonication pretreatment on the total polyphenol extraction yield from elderberry flowers (EBF), using stirred-tank extraction under optimal conditions, at 80 °C. Ultrasonication prior to stirred-tank extractions was performed at ambient temperature (22 ± 1 °C). Asterisk (*) indicates statistically different value (*p* < 0.05).

The pretreatment consisted of ultrasonicating the samples prior to performing batch-stirred tank extraction under optimized conditions, at 80 °C. Ultrasonication was carried out for a period varying from 5 to 40 min at ambient temperature (23 ± 1 °C), and the results are portrayed in Figure 5. After the ultrasonication step, the Y_TP_ was, at best, almost 50% lower than that achieved with the stirred-tank extraction. This finding strongly emphasized that ultrasonication is ineffective as a standalone extraction methodology, which is in accordance with previous observations [12,14], although contradictory results have also been reported [36]. However, when ultrasonication was accompanied by stirred-tank extraction, Y_TP_ determined was always significantly higher than that attained without ultrasonication pretreatment. It was also notable that Y_TP_ displayed statistically non-significant variations as a response to ultrasonication time. Thus, even the shortest ultrasonication period tested (5 min), resulted in a very important enhancement of the yield after 150 min of stirred-tank extraction. This is in line with recent kinetic data on the extraction of polyphenols from hop (*Humulus lupulus*) using a glycerol/L-alanine DES and ultrasonication as a pretreatment step, which evidenced significant enhancement of subsequent stirred-tank extraction, at 80 °C [13].

Irradiation with ultrasound is known to intensify solid–liquid extraction through generation of cavitation effects [37]. The collapse of cavitation bubbles nearby or on the surface of the solid particles is considered to cause particle disruption and destruction of cell walls, as well as intense shaking at a macroscopic level (ultrasound streaming), which may contribute in fast washing of the superficial solute, solvent penetration into canals and pores of plant material, and eventually increased diffusivity, high entrainment of the solute into the liquid phase, and enhanced solubilization. All these phenomena may be responsible for increasing polyphenol extraction yield [11].

### 3.5. Polyphenolic Composition

**Figure 6:**
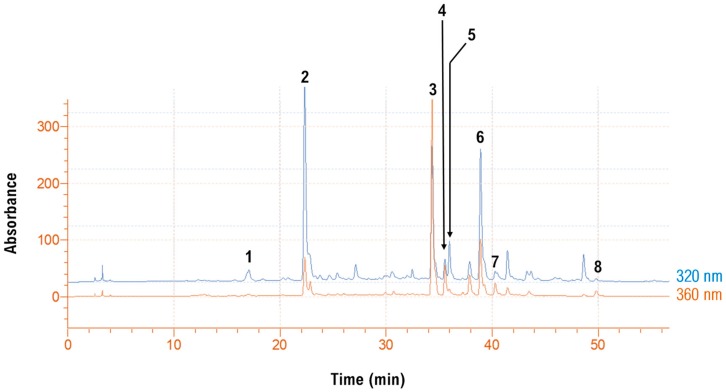
HPLC traces of the extract obtained with 10-min ultrasonication pretreatment, under optimal conditions, with LA-Gly (5:1). Traces were recorded at 320 (hydroxycinnamates) and 360 (flavonols) nm.

The richest EBF extract was produced with a 10 min ultrasonication pretreatment and then stirred-tank extraction under optimized conditions, at 80 °C, for 150 min (Figure 5). This sample was chosen to profile its analytical polyphenolic composition, and the trace recorded at both 320 and 360 nm revealed the presence of several chlorogenate and flavonol derivatives (Figure 6). By carrying out liquid chromatography–diode array–mass spectrometry analysis, it was made possible to tentatively identify eight polyphenolic compounds (Table 4). A total ion chromatogram is also provided (Figure S1). Concerning chlorogenates, peak #1 showed a pseudo-molecular ion at *m*/*z* = 355 and a diagnostic fragment at *m*/*z* = 163. Considering the retention time of the original standard, this compound was tentatively identified as neochlorogenic acid. In a similar fashion, peak #2 was identified as chlorogenic acid [14]. Peak #5 displayed a pseudo-molecular ion at *m*/*z* = 517 and two fragment ions at *m*/*z* = 355 and 163. This structure was assigned to a di-caffeoylquinic acid [38]. Peak #6 gave a pseudo-molecular ion at *m*/*z* = 485, and a diagnostic fragment at *m*/*z* = 147. This compound was identified as a di-*p*-coumaroylquinic acid derivative [39].

With regard to flavonols, peak #3 yielded a pseudo-molecular ion at *m*/*z* = 611 and fragment ion at *m*/*z* = 303. These data, along with the retention time of the original standard, enabled the identification of this substance as rutin (quercetin 3-*O*-rutinoside).

Likewise, peak #8 was identified as quercetin. Peak #4 gave a pseudo-molecular ion at *m*/*z* = 465 and fragment ion at *m*/*z* = 303, which pointed to the structure of quercetin 3-*O*-glucoside (isoquercitrin). For peak #7, a pseudo-molecular ion was detected at *m*/*z* = 625, an adduct with Na^+^ at *m*/*z* = 647 and a diagnostic fragment at *m*/*z* = 317. This structure was tentatively assigned to isorhamnetin 3-*O*-rutinoside (narcissin) [40].

On the basis of the quantitative analysis, the predominant constituents were rutin (17.36 mg g^−1^ dm), di-*p*-coumaroylquinic acid (13.06 mg g^-1^ dm), and chlorogenic acid (10.76 mg g^−1^ dm) (Table 5).

According to a survey on flower composition of 16 different *S. nigra* genotypes [41], the average content of neochlorogenic acid, chlorogenic acid, rutin and isoquercitrin were 1.6, 15.2, 21.0, and 1.0 mg g^−1^ dm, respectively. For neochlorogenic acid, chlorogenic acid, rutin, isoquercitrin and narcissin, corresponding content ranges were shown to be 1.06–1.60, 12.40–14.00, 15.70–23.90, 0.73–3.05, and 4.26–5.33 mg g^−1^ dm [42]. Data from another study on EBF extracts were in line, reporting contents for chlorogenic acid, rutin, isoquercitrin, and quercetin of 5.93, 15.28, 2.64, and 0.11 mg g^−1^ dm [43]. Rutin and isoquercitrin contents of 20.2 and 0.97 mg g^–1^ have also been reported, in EBF extracts obtained with pressurized liquid extraction [44]. The values reported herein are close to these levels. On the other hand, microwave- and ultrasound-assisted extraction of EBF with 50% ethanol has been reported to give contents for chlorogenic acid and rutin of 56.49 and 91.39 mg g^−1^ dm, respectively [45].

## 4. Conclusions

The use of an effective DES, composed of lactic acid and glycine, along with the implementation of an appropriate experimental design, allowed for a high-performance extraction of polyphenols from EBF. The temperature assay showed that even higher extraction yield may be achieved by carrying out extraction up to 80 °C, obtaining extracts with improved antioxidant properties. The integration of ultrasonication as a pretreatment step, enabled the production of EBF extracts enriched in polyphenols. It was also demonstrated that even a ultrasonication regime of 5 min may significantly boost the yield of subsequent stirred-tank extraction. Extract characterization with liquid chromatography–mass spectrometry revealed that EBF extracts were dominated by chlorogenic acid, a di-*p*-coumaroylquinic acid and rutin. As a general conclusion, it could be argued that combination of the DES used with ultrasonication pretreatment may afford exceptionally high extraction yields in polyphenols, yet safety issues regarding EBF extracts remain to be clarified by future studies. The advantages of the methodology proposed remain to be tested by comparison with other green techniques.

## Figures and Tables

**Table 1:** Actual and codified levels of the variables used for the design of experiment.

Independent Variables	Code Units	Coded Variable Level		
		−1	0	1
*C*_DES_ (%, *w*/*v*)	X_1_	55	70	85
R_L/S_ (mL g^−1^)	X_2_	20	40	60
S_S_ (rpm)	X_3_	200	500	800

**Table 2:** Statistical information related with model fitting derived from response surface methodology.

Term	Standard Error	t Ratio	Probability > t	Sum of Squares	*F* Ratio
Intercept	1.666545	57.83	< 0001 *	31.08661	3.7309
*C*_DES_	1.020546	1.93	0.1113	124.7410	14.971
R_L/S_	1.020546	3.87	0.0118 *	185.6664	22.283
S_S_	1.020546	−4.72	0.0052 *	17.22250	2.0670
*C*_DES_ R_L/S_	1.443270	−1.44	0.2100	10.33622	1.2405
*C*_DES_ S_S_	1.443270	−1.11	0.3160	4.182020	0.5019
R_L/S_ S_S_	1.443270	0.71	0.5103	74.05096	8.8874
*C*_DES_ *C*_DES_	1.502204	2.98	0.0308 *	155.9200	18.713
R_L/S_ R_L/S_	1.502204	4.33	0.0075 *	147.9857	17.761
S_S_ S_S_	1.502204	4.21	0.0084 *	31.08661	3.7309
Lack-of-fit			0.0817	39.3593	11.402

Asterisk (*) indicates statistically different value (*p* < 0.05).

**Table 3:** Measured and predicted values of the response for all design points considered.

Design Point	Independent Variables			Response (Y_TP_, mg GAE g^−1^ dw)	
	X_1_ (*C*_DES_, % *w*/*v*)	X_2_ (R_L/S_, mL g^−1^)	X_3_ (S_S_, rpm)	Measured	Predicted
1	−1 (55)	−1 (20)	0 (500)	100.43	100.87
2	−1 (55)	1 (60)	0 (500)	112.48	112.92
3	1 (85)	−1 (20)	0 (500)	106.39	105.95
4	1 (85)	1 (60)	0 (500)	110.14	109.70
5	0 (70)	−1 (20)	−1 (200)	109.16	109.60
6	0 (70)	−1 (20)	1 (800)	101.37	100.93
7	0 (70)	1 (60)	−1 (200)	115.01	115.45
8	0 (70)	1 (60)	1 (800)	111.31	110.87
9	−1 (55)	0 (40)	−1 (200)	109.31	108.43
10	1 (85)	0 (40)	−1 (200)	118.60	118.60
11	−1 (55)	0 (40)	1 (800)	99.00	102.01
12	1 (85)	0 (40)	1 (800)	101.86	102.74
13	0 (70)	0 (40)	0 (500)	97.56	96.38
14	0 (70)	0 (40)	0 (500)	95.46	96.38
15	0 (70)	0 (40)	0 (500)	96.13	96.38

**Table 4:** Spectral information for the principal polyphenolic constituents tentatively identified in the EBF extract obtained under optimal conditions, at 80 °C.

No	Rt (min)	UV-Vis (λ_max_)	[M + H]^+^ (m/z)	Other Ions (*m*/*z*)	Tentative Identity
1	17.22	328	355	163	Neochlorogenic acid
2	22.4	328	355	163	Chlorogenic acid
3	34.32	255, 352	611	303	Quercetin 3-*O*-rutinoside (rutin)
4	35.50	254, 352	465	303	Quercetin 3-*O*-glucoside (isoquercitrin)
5	35.97	318	517	355, 163	Di-caffeoylquinic acid
6	38.90	318	485	147	*p*-Coumaroylquinic acid derivative
7	40.28	351	625	647[M + Na]^+^, 317	Isorhamnetin 3-*O*-rutinoside (narcissin)
8	49.4	259, 369	303	-	Quercetin

**Table 5:** Quantitative data for the principal polyphenols tentatively identified in the EBF extract, obtained under optimal conditions, at 80 °C.

Polyphenol	Content (mg g^−1^ dm) ± SD
Phenolic acids	
Neochlorogenic acid	1.11 ± 0.01
Chlorogenic acid	10.76 ± 0.45
Di-caffeoylquinic acid	2.55 ± 0.04
di-p-Coumaroylquinic acid derivative	13.06 ± 0.89
Total	27.48
Flavonols	
Quercetin 3-O-rutinoside (rutin)	17.36 ± 1.10
Quercetin 3-O-glucoside (isoquercitrin)	2.06 ± 0.05
Isorhamnetin 3-O-rutinoside (narcissin)	0.96 ± 0.00
Quercetin	0.53 ± 0.00
Total	20.91
Sum	48.39

## Supplementary Materials

The following are available online, Figure S1: Total ion chromatogram showing all peaks tentatively identified in the EBF extract obtained under optimized conditions, at 80 °C.

## Abbreviations

A_AR_	antiradical activity (μmol DPPH g^−1^)
P_R_	reducing power (μmol AAE g^−1^)
R_L/S_	liquid-to-solid ratio (mL g^−1^)
*t*	time (min)
*T*	temperature (°C)
Y_TFn_	yield in total flavonoids (mg RtE g^−1^)
Y_TP_	yield in total polyphenols (mg GAE g^−1^)
AAE	ascorbic acid equivalents
DPPH	2,2-diphenyl-1-picrylhydrazyl radical
GAE	gallic acid equivalents
HBA	hydrogen bond acceptor
HBD	hydrogen bond donor
NADES	Natural deep eutectic solvents
TPTZ	2,4,6-tripyridyl-*s*-triazine
