# Supplementary material for: A Green Extraction Process for Polyphenols from Elderberry (Sambucus nigra) Flowers Using Deep Eutectic Solvent and Ultrasound-Assisted Pretreatment
Source: Molecules. 2020 Feb 19;25(4):921. doi: 10.3390/molecules25040921 (PMC7070494; doi:10.3390/molecules25040921)
Supplement: Supplementary file 1 [file molecules-25-00921-s001.pdf]

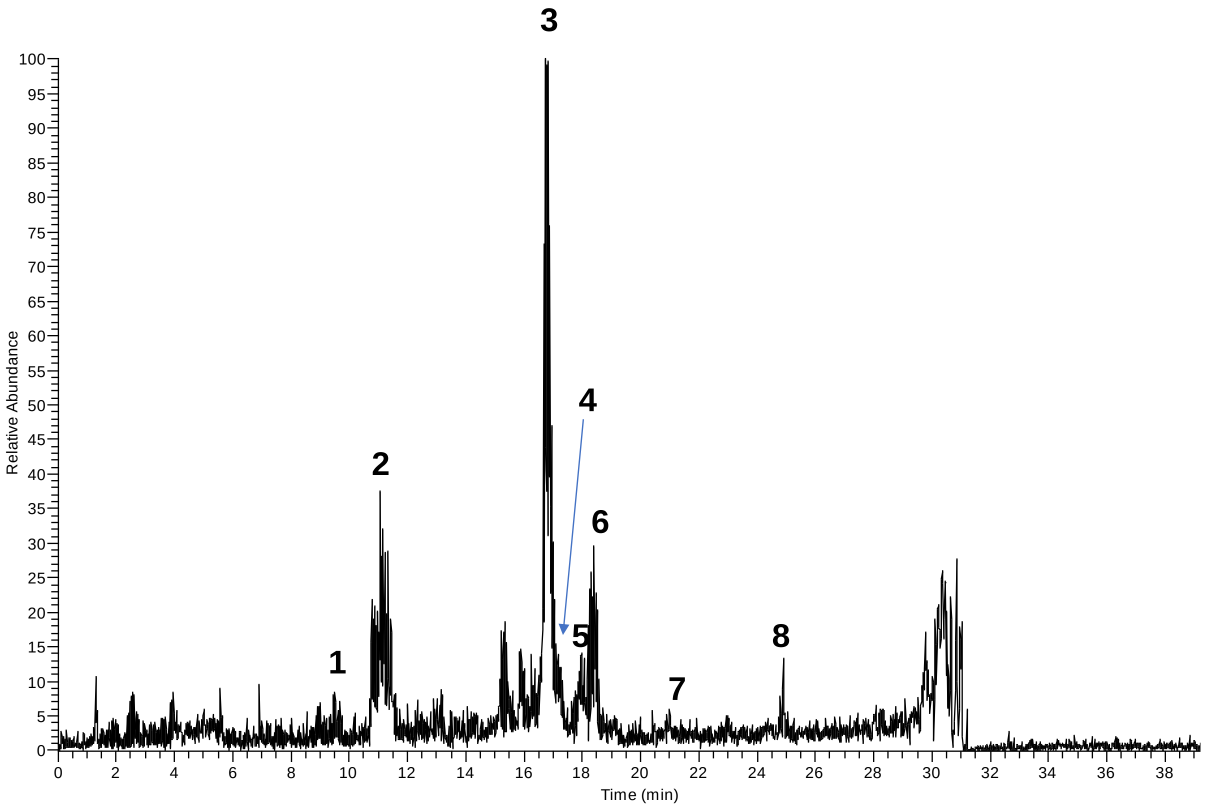


**Figure S1**: Total ion chromatogram showing all peaks tentatively identified in the EBF extract obtained under optimized conditions, at 80 °C. For peak assignment, see Table 4.
